# Crafting your scientist brand

**DOI:** 10.1371/journal.pbio.3000024

**Published:** 2018-10-05

**Authors:** Peter J. Hotez

**Affiliations:** 1 Texas Children’s Hospital Center for Vaccine Development, Department of Pediatrics, National School of Tropical Medicine, Baylor College of Medicine, Houston, Texas, United States of America; 2 Texas Children’s Hospital Center for Vaccine Development, Department of Molecular Virology and Microbiology, National School of Tropical Medicine, Baylor College of Medicine, Houston, Texas, United States of America; 3 Department of Biology, Baylor University, Waco, Texas, United States of America; 4 James A. Baker III Institute of Public Policy, Rice University, Houston, Texas, United States of America; 5 Scowcroft Institute of International Affairs, Bush School of Government and Public Policy, Texas A&M University, College Station, Texas, United States of America

## Abstract

That a scientist might shape and cultivate a personal brand is a relatively new concept but one that is finding increasing acceptance in this new age of rapid communications and social media. A key driver is the abrupt rise in well-funded and organized antiscience movements, especially in North America and Europe, such that society now benefits from scientists with strong personal brands and public personas who are willing to engage general audiences. In this sense, branding itself can advance science, the sharing of information, and the promotion of science as a public good. Still another dimension to branding is that it affords an opportunity to mentor younger scientists and helps you to become an important role model for the next generation. There is also a practical side, as today, fewer scientists spend their entire career at a single institution, so owning a strong brand can sometimes create easier paths for transitions and mobility. However, brand cultivation ideally begins in collaboration with your institutional office of communications and is done in a way that is seen as a win for both you and your university or research institution. Described here are some steps to consider when embarking on brand cultivation and how to avoid some of the potential pitfalls.

## Introduction to the personal brand

We typically think of a brand as the distinctiveness of a specific product or organization for purposes of marketing or advertising. McDonald’s, the iPhone, and Google are each internationally recognized brands. But I only began to think deeply about my personal brand in 2012, about a year after relocating to Baylor College of Medicine and Texas Children’s Hospital in Houston, Texas. Our basketball team, the Houston Rockets, was interested in acquiring a new center, Dwight Howard, from the Los Angeles Lakers. At the time, losing Dwight Howard to free agency was a big loss for the Lakers, but when their star player, Kobe Bryant, was asked about it, he graciously offered [1], “I'm happy for him. It's important for free agents to make decisions that they feel [are] best for them. That's really what it's about, being a free agent. You have to make decisions that you feel is best for you, best for your family and best for your brand, whatever it may be. So, it is what it is”.

**Fig 1:**
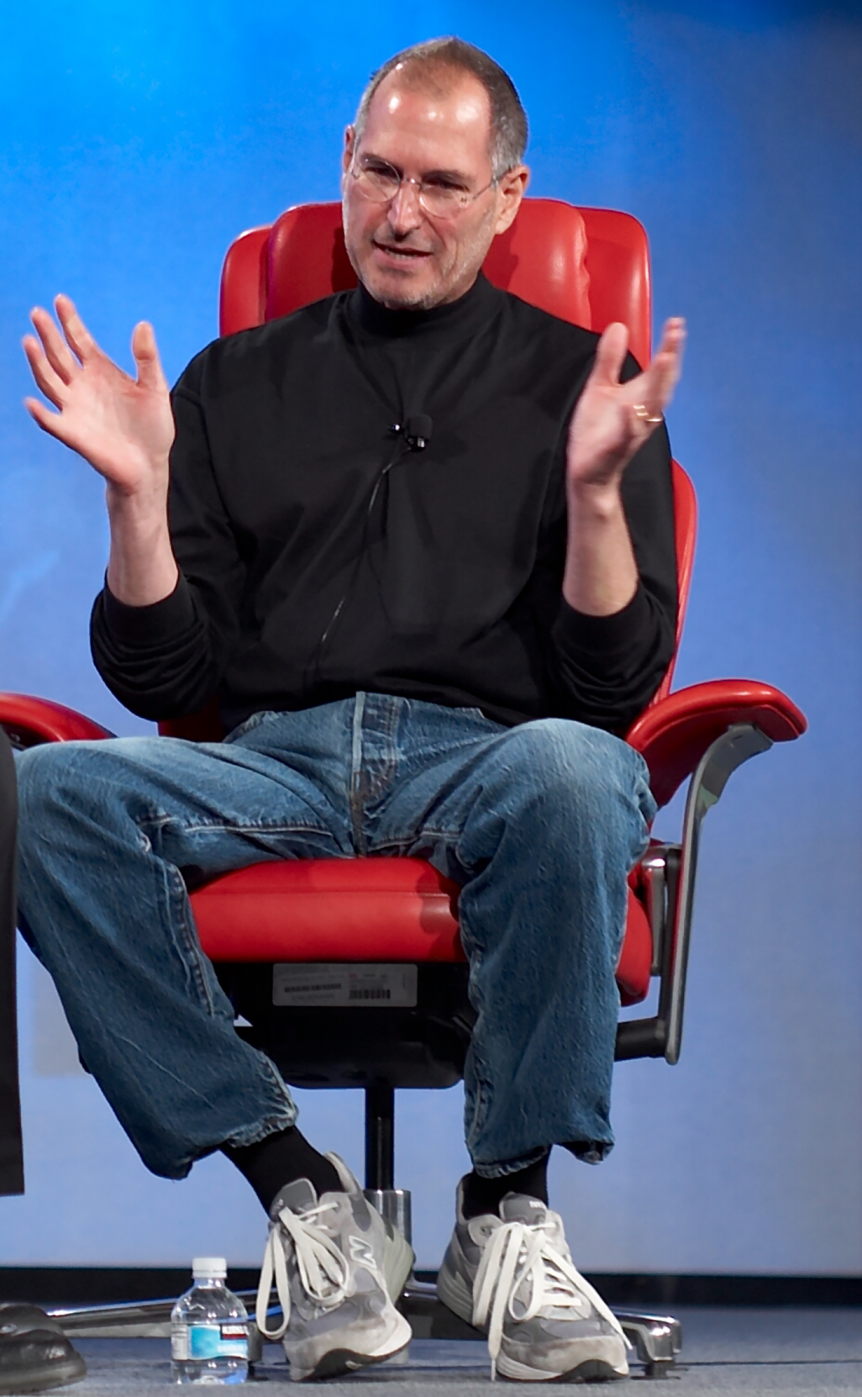
Steve Jobs. Cropped from photo by Flickr user Joi, https://www.flickr.com/photos/joi/522218512/sizes/o/in/photostream/.

That’s when the potential for personal brands really hit home. Indeed, the corporate strategist Robert Holtz points out that some of our best-known corporations and other organizations are led by individuals with strong and even iconic personal brands [2]. One of the examples he cites is Steve Jobs’ 1990s style of “St. Croix black mock turtlenecks, Levi 501 blue jeans, and New Balance sneakers” (Fig 1) [2], but of course, personal brands can go way beyond just clothes to reflect a unique persona and authentic image. Holtz goes on to explain the essential elements of a person as a brand, including the importance of being memorable or standing out, offering a key message, and the importance of authenticity or “walking the walk” in addition to “talking the talk,” as he points out [2]. Today, many, if not most, high-profile authors, entertainers, and people in the arts cultivate a personal brand [2].

### Scientists

So what about scientists? Should scientists attempt to craft their brand? Well, back in the 1980s, when I was an MD–PhD student at Rockefeller University and Weil Cornell Medical College, the answer was a pretty resounding “no.” In fact, scientists who strayed in that direction by cultivating relationships with journalists or even engaging the public directly were often derided as self-promoters or grandstanders. Indeed, Paul de Kruif, the author of *Microbe Hunters* [3], a book that influenced and inspired a generation of young people going into science (including me), was allegedly fired from the Rockefeller Institute of Medical Research (the founding name of today’s Rockefeller University) because he contributed an article about the medical profession for the popular reader. Perhaps in retaliation, he served as a science advisor to Sinclair Lewis for his great American novel *Arrowsmith* [4], in which de Kruif provided detailed descriptions of scientists for Lewis’ fictitious McGurk Institute in New York, which, in many cases, were really thinly veiled portrayals of Rockefeller Institute scientists.

Times have changed, and I argue here that it has become increasingly helpful and sometimes even necessary for at least some scientists to cultivate a personal brand. I can think of at least two major reasons for which branding might be a real advantage for a scientist, especially a young scientist.

### Brands move with you

In the United States (and increasingly internationally), it’s no longer the case that most biomedical scientists are employed by a single institution for their entire career. There are a number of reasons for this, including the need to change institutions in order to advance, especially for scientists working at academic health centers. In turn, many academic health centers are becoming less and less stable due to shifts in leadership, science funding, and healthcare dollars. For those reasons, defining your academic or scientific identity solely on the basis of your current position and title today can represent a high-risk strategy, whereas brand cultivation can facilitate smoother career transitions.

### The world, but especially America, needs brand name scientists

In this age of social media and 24-hour news, our scientific voice is rapidly disappearing. In January 2018, the policy and advocacy think tank Research!America released a new poll, finding that 81% of Americans cannot name a living scientist [5]. Moreover, those 19% who could name a scientist mentioned people of great stature and importance such as Bill Nye and Richard Dawkins but not necessarily individuals who still struggle over grants and papers and spend large parts of their days in laboratory meetings. Especially left out in terms of recognition are the science graduate students, postdocs, research associates, and staff associates [6]. In other words, the American public is mostly unfamiliar with scientists who wake up in the morning and head off to their laboratories or computer work stations.

Yet another study conducted by the Pew Research Scientist found that only a small minority of scientists blog about their work or use social media to discuss or follow science [7]. My takeaway is that the general public overall has a very poor understanding of what a working scientist actually does. This absence of knowledge about our daily activities is partly our fault—as scientists, we’re so focused on our grants and papers and writing (and speaking) for each other that we’re losing touch with the general public. While studies conducted by the American Academy of Arts and Sciences suggest that confidence in American scientific leadership remains relatively stable and robust [8], there have been some worrisome increases in antiscientific trends, such as a rising antivaccine movement and denials in the evidence for climate change and the impact of rising gun violence [9]. I often wonder how much the rise in recent antiscience trends has occurred in the context of this vacuum in science public engagement. To begin countering this trend, we need more scientists out there in the public domain who are willing to develop a brand and engage a generally bewildered populace.

For me, the essential elements to cultivate a brand include a self-awareness of who you are, what you wish to project to the public, and, most importantly, what major problem you want to solve and what you want to achieve through your science. Cultivating a brand as a vaccine pediatrician scientist committed to developing neglected disease vaccines and providing the world’s poor access to innovation [10–12] has allowed me to partly achieve those objectives. My brand has helped to give me the mobility I’ve needed to establish a new school of tropical medicine and vaccine research center in Texas, and as both a vaccinologist and autism dad, it’s helped in my struggle to combat a well-organized and well-funded pseudoscience antivaccine lobby whose American epicenter is now also based here in Texas [13].

### Role models for the next generation

**Fig 2:**
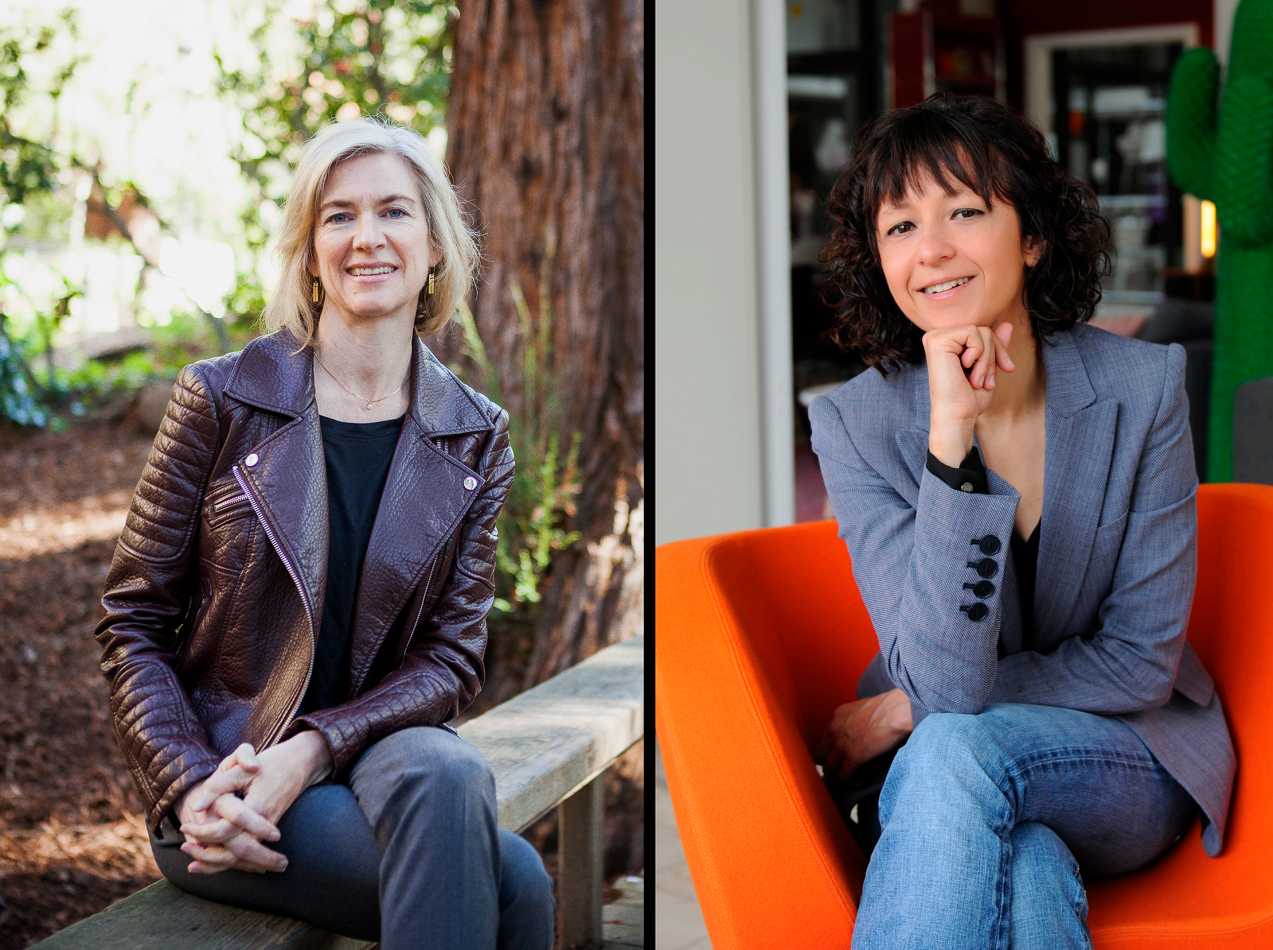
Jennifer A. Doudna (left) and Emmanuelle Charpentier (right), winners of the 2015 Breakthrough Prize. *Dr*. *Doudna picture by Flickr user Jussi Puikkonen/KNAW*, *https*:*//www*.*flickr*.*com/photos/79173061@N08/26658739920/**; Dr*. *Charpentier picture courtesy of Hallbauer + Fioretti*, *Braunschweig (Germany)*.

This is an exciting time for scientists to craft their brand, in part because there is a rising new generation of strong and diverse role models. For instance, the Breakthrough Prize in Life Sciences has given urgently needed high visibility to several prominent women scientists, such as Joanne Chory (Salk Institute), Huda Zoghbi (Baylor College of Medicine), Helen Hobbs (University of Texas Southwestern), Jennifer Doudna (University of California–Berkeley), Emmanuelle Charpentier (Max Planck Institute), and Cornelia Bargmann (Rockefeller University) (Fig 2) [14], just to name a few. And now we’re also seeing highly visible junior scientists, including important African American and Hispanic women in science, technology, engineering, and mathematics (STEM) fields [15,16].

### Crafting a brand

I believe that success in brand cultivation relies initially on focusing your writings and public engagement and not trying to be everything to everyone. In that respect, the Massachusetts Institute of Technology (MIT) physicist and writer Alan Lightman identifies three tiers of public intellectuals, each requiring a certain level of branding. He lists them as follows [17]:

*Level I: Speaking and writing for the public exclusively about your discipline*.

*Level II: Speaking and writing about your discipline and how it relates to the social, cultural, and political world around it*.

*Level III: By invitation only. The intellectual has become elevated to a symbol, a person that stands for something far larger than the discipline from which he or she originated*.

By all means, start at Level I and build out, accordingly. It’s never too early to begin brand cultivation, even as a graduate student or postdoctoral fellow, and you can begin modestly and incrementally (see Box 1). The tactics involve writing commentaries in biomedical journals that allow them, as well as writing relevant op-eds and blogs in magazines, newspapers, and websites. Blogging is a great place to begin. Delivering lectures to general scientific audiences but also lay audiences is also an important component, as is creating a personal website and cultivating a social media presence, for example, on Twitter. It’s interesting to note that most of the leading science journalists are active on Twitter. Countless times, I’ve received a call or e-mail from journalists that begin, “I saw your tweet yesterday…” Indeed, taking the time to cultivate relationships with journalists is an important component. Still another dimension to branding is that it affords an opportunity to share information and mentor younger scientists. It can help you become an important role model for the next generation.

**Box 1. Cultivating a brand**

- Focus your writings and public engagement around your self-awareness of who you are, what you want to project to the public, and what important problem you want to solve and achieve through your science:
  ○ Writing commentary pieces in leading biomedical journals
  ○ Writing relevant op-eds/blogs in magazines, newspapers, or websites
  ○ Writing a book (or multiple books)
  ○ Delivering grand rounds presentations, university lectures, TED Talks, and presentations at national meetings
  ○ Social media presence (Twitter)
  ○ Creating a personal website
  ○ Cultivating relationships with journalists, both print and electronic
  ○ Press releases for key scientific papers
- Encourage and mentor others.
- Work with your institutional office of communications to get help with shaping your brand and avoiding potential minefields.

### Proceeding thoughtfully

There is also a downside to brand cultivation. Unfortunately, branding is still not in the DNA of our profession, so don’t expect your principal investigator, department chair, or dean to necessarily or immediately embrace your public outreach. This is especially true at academic health centers, which are still heavily focused on revenue-generating mechanisms and for which blogs, op-eds, personal websites, and social media generally defy any traditional metric used for advancement and promotion. If you decide to take the route of cultivating a personal brand, it’s often helpful to discuss your plans beforehand with your institutional office of communications. They are typically comprised of professionals with lots of experience in working with the print and electronic media who can provide helpful suggestions as well as prepare you for possible mine fields or other obstacles. They can also help draft press releases around important scientific articles that you’ve written and published. Developing a relationship with your institutional communications office will also serve you well down the line in the event of a misstep or negative public reaction against something you’ve said or written.

Branding also means personal exposure, which can require courage when that involves going up against antiscience movements that deploy harassment and intimidation tactics on social media. It helps to have a strong sense of self, not to mention a sense of humor and humility. In the end, branding should be an activity that you find fun and meaningful. If it turns out to be just one more source of stress in an already stressful profession, you can always step away from it.

Despite such obstacles, I believe that brand cultivation will become an essential ingredient for success in 21st century science, and one that may become essential to ensure the survival of our profession. In this sense, branding itself can advance science, the sharing of information, mentoring, and the promotion of science as a public good.

## Abbreviations

MIT: Massachusetts Institute of Technology
STEM: science, technology, engineering, and mathematics
